# Retraction Note: Human placental extract rescues hippocampal damage associated with cognitive impairment in diabetic male rats through antioxidative, anti-inflammatory, and neuromodulatory activities

**DOI:** 10.1007/s11011-026-01806-y

**Published:** 2026-02-07

**Authors:** Shreen Matar, Rehab A. Gomaa, Abeer El Wakil, Amina Essawy

**Affiliations:** 1 Department of Zoology, Faculty of Science, Alexandria University, Alexandria, 21511 Egypt; 2 Department of Biological and Geological Sciences, Faculty of Education, Alexandria University, Alexandria, 21526 Egypt

**Retraction Note: Metabolic Brain Disease (2025) 40:225**

10.1007/s11011-025-01646-2

The Editor-in-Chief has retracted this article.

Following publication, concerns have been raised regarding partial image overlaps in Comet Assay presented in Figs. 5A, 5B and 5C. The authors stated that these experiments were outsourced from a third party and were unable to provide raw images for the concerned Figure. Additionally, Figs. 5G and 5H were found to be identical due to an error in figure preparation.

The Editor-in-Chief therefore has lost confidence in this data.

Abeer El Wakil stated that all authors disagree with this retraction.

